# The eggshell structure in *apteryx*; form, function, and adaptation

**DOI:** 10.1002/ece3.7266

**Published:** 2021-02-26

**Authors:** David Vieco‐Galvez, Isabel Castro, Patrick C. H. Morel, Wei Hang Chua, Michael Loh

**Affiliations:** ^1^ School of Agriculture and Environment Massey University Palmerston North New Zealand; ^2^ School of Health Sciences Massey University Palmerston North New Zealand; ^3^ Fonterra ltd. Palmerston North New Zealand

**Keywords:** Apteryx, embryonic gas exchange, incubation physiology, Kiwi eggs, water vapour conductance

## Abstract

*Apteryx* is a genus of flightless birds endemic to New Zealand known to lay very large eggs in proportion to body weight. The eggshell of *Apteryx* is unusually thin and less porous than allometrically expected possibly as a compensation for a very long incubation period. Past studies have been carried out on *Apteryx australis,* a species which once comprised all kiwi with brown plumage, now separated into three distinct species. These species use different habitats and live at different latitudes and altitudes, therefore generating a need to revise our knowledge of the attributes of their eggshells. In this study, we measured the physical characteristics and water conductance on eggshell fragments of these three species and Great‐spotted Kiwi and relate them to the environmental conditions of their respective environments; we also measured the water vapor conductance of Brown Kiwi eggs of late stages of incubation. We found that several trade‐offs exist between incubation behavior, environmental conditions, and eggshell structure. We found differences between species in eggshell water vapor conductance seemingly related to altitude; Brown Kiwi and Rowi generally inhabiting lower altitudes had the highest conductance and Tokoeka, generally living in montane environments, the lowest. This is achieved by an increased eggshell thickness rather than a pore area reduction. Finally, the water vapor conductance late in incubation was 58% higher than infertile unincubated eggs, suggesting a drastic increase in conductance throughout the long incubation period. Using the values previously reported, we calculated the embryonic eggshell thinning to be 32.5% at the equatorial region of the eggshell. We describe several new features, such as triangular mineral particles in the cuticle, reported for the extinct *Trigonoolithus amoei*, and confirmed the existence of plugged pores. We suggest that these structures provide microbial protection needed by a burrow nesting species with a long incubation period.

## Graphical abstract

Apteryx incubates its eggs for 74 days in a ground burrow. The eggshell structure reveals adaptations to this type of environment, and in relation to the climate, the eggshell has been laid in.

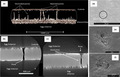

## 1 INTRODUCTION

Birds reproduce successfully in a wide range of ecosystems, including some of the most inhospitable ones (e.g., extreme cold, deserts, compost mounds, high altitude). This is possible because birds have evolved delicately balanced strategies to ensure the survival of their eggs (Carey, 1980a). Species are able to fine tune their physiology (Drake et al., 2017), anatomy (Wright et al., 2016), and behavior (Forstmeier & Weiss, 2004) to respond to climatic regimes, predation, parasitism, and intraspecific competition. This is why such variety exists in the physical characteristics of eggs and eggshells (Mikhailov et al., 1996; Portugal et al., 2014), nest architecture (Warning & Benedict, 2015), and incubation behaviors (Deeming, 2002) of egg laying vertebrates. The relationships that exist between these adaptations and environmental drivers mean that using the characteristics of eggs, we could better understand the nesting ecology of species (Tanaka et al., 2015). For example, several studies have hypothesized the nesting ecology of extinct species based on the physical characteristics of their fossilized eggshells (Deeming et al., 2006; Grellet‐Tinner et al., 2006; Varricchio et al., 2013) and through comparisons of the physical characteristics of their fossilized eggshells with those of close extant relatives. In addition, studying the physical characteristics of eggs and eggshells of extant species allows to piece together the relationship between nesting ecology, eggshells characteristics (adaptations), and successful breeding.

The avian eggshell is a bioceramic composed mainly of calcium carbonate organized in four mineral layers; additionally, there are two inner proteinaceous membranes (Romanoff & Romanoff, 1949); the shell as a whole allows the gas exchange with the external environment through passive diffusion (Rahn & Paganelli, 1990). The cuticle, the usually pigmented outermost layer of the shell, has been proposed to have antimicrobial properties (D’Alba et al., 2014), UV wavelength modulation properties, (Cooper et al., 2011), and water repellence. The latter has been found to be strongly associated with prevention against waterborne bacterial penetration (Sparks & Board, 1984). These characteristics suggest that the cuticle characteristics are affected by environmental conditions such as solar radiation and pluviosity, but also to the nest environment, mainly the probability of flooding and the potential for bacterial contamination (D’Alba et al., 2014, 2017).

The thickness of the eggshell is a functional character associated with reduction of bacterial infection as a solid barrier (Board & Fuller, 1974; D’Alba et al., 2014, 2016, 2017) and gas exchange as the eggshell is a porous material (Rahn & Ar, 1974). Most avian species present funnel like pores; however, simple branched pores have been described in Emperor Penguin (*Aptenodytes forsteri*), Wandering Albatross (*Diomedea exulans*), and various species within Anseriformes (mostly Swans). Branched pores in the longitudinal axis of the egg have been seen in the eggshells of Rhea (*Rheiformes*), Moa (Dinornithiformes), and Elephant Birds (Aepyornithiformes), and branched pores that extend in all planes are present in Ostriches (*Struthio camelus*). Finally, a combination of single and fork‐like pores has been described in Emu (Dromaius novaehollandiae) and Cassowary (Casuariiformes) (Tullett, 1978). It could be said that branched pores are common among ratites with Tinamou (Tinamiformes) and Kiwi being the exception. The number and shape of pores are related to the incubation period (Zimmermann et al., 2007), with less porous eggs being associated with longer incubation periods; altitude where the birds usually live, with porosity decreasing at altitudes (Rahn et al., 1977a); and the nest microclimate usually increasing in porosity in high humidity environments (Birchard & Kilgore, 1980). An additional contributor to eggshell thickness is the bioavailability of calcium, and it is mainly obtained through diet (Wilkin et al., 2009). Furthermore, the embryo extracts calcium from the mammillary layer of the eggshell for skeletal development (Blom & Lilja, 2004) making the eggshell progressively thin, and this is more prominent in precocial species being up to 7.3% at the equator, while for altricial species is about 5.7% (Orłowski & Hałupka, 2015). This eggshell thinning during incubation increases water vapor conductance.

Eggshell porosity is a function of the pore functional area (mean area of individual pores multiplied by the total number of pores in an eggshell) and the thickness of the eggshell (Board & Scott, 1980). These two characteristics of the eggshell in conjunction with the environment of the nest and the properties of the cuticle, if present, determine the gas conductance of the eggshell (Peebles & Brake, 1986). Gas conductance is a measurement of the gas transfer through a medium (Ar et al., 1974; Rahn & Ar, 1980; Rahn & Paganelli, 1990). Gas exchange allows the necessary oxygen for embryo development to diffuse into the egg and allows carbon dioxide and water vapor to leave the egg (Maina, 2017; Mueller et al., 2014). This poses a series of constraints for different species incubating in different environmental conditions (Carey, 1980). Birds incubating in dry environments need to retain more water to avoid desiccation (Grant, 1982), while species in wet environments need to increase the water vapor conductance to lose enough water for the embryo to develop, not losing enough water can result in respiratory problems while hatching (Deeming, 2011; Maina, 2017).

Altitude is an important factor in regulating water vapor conductance due to the differences in barometric pressure that affect the rate of gas diffusion (Rahn et al., 1977a, 1977b). Therefore, it is expected that different species will present particular adaptations to regulate the water loss in different habitats and use different incubation techniques and nesting behaviors (Birchard & Kilgore, 1980; Portugal et al., 2010; Whittow et al., 1987).

Burrow nesting species represent an extreme example in terms of adaptations to cope with high humidity, low concentrations of oxygen, and high concentrations of carbon dioxide (Boccs et al., 1984; Collias, 1986). Therefore, it is generally expected that burrowing species have an increased water vapor conductance to allow sufficient water loss and oxygen intake. In turn, burrow nesting may improve egg survivorship by providing better protection from predators and/or a more constant environment for embryo development (Boccs et al., 1984; Boggs & Kilgore, 1983; Whittow et al., 1987).

The Apterygidae is a family of nocturnal, flightless birds endemic to New Zealand, and it is characterized by a series of unique traits that are rare or not present in any other clade of birds, including other ratites (Ramstad & Dunning, 2020). Among these traits are the egg's size, which is large in relation to bird size (Calder, 1979; Taborsky & Taborsky, 1999), comprising between 14%–23% of the female's body weight (Dyke & Kaiser, 2010), the very thin eggshell compared to the size of the egg, which is 27% thinner than allometrically expected (Calder, 1979), and the use of a burrow nest (Colbourne, 2002; Jolly, 1989; Vieco, 2019). *Apteryx* species are precocial, meaning that the chicks hatch fully feathered and almost ready to eat by themselves (Vleck et al., 1979).

The genus *Apteryx* contains five well‐defined species distributed in the three main Islands of New Zealand and some offshore islands (Burbidge et al., 2003; Weir et al., 2016). *Apteryx* species present a very localized distribution due to the decline of their natural populations as a result of predation by introduced mammals and loss and fragmentation of habitat by deforestation (Germano et al., 2018). The climate encountered by *Apteryx* species varies from mild temperatures in the north of New Zealand to below zero temperatures and snow in the south (www.worldweatheronline.com).

*Apteryx* nests in globular cavities dug in the ground or existing cavities in dead trees or tree roots (Ziesemann et al., 2011) lined with nesting materials (Vieco, 2019). And in Brown Kiwi, the eggs are partially buried in the nest lining, which can influence eggshell gas conductance (Colbourne, 2002). *Apteryx* is an unusual ratite as it has evolved to be mostly entirely nocturnal and primarily insectivorous (Cunningham & Castro, 2011; Le Duc et al., 2015).

Finally, *Apteryx* eggs are incubated for approximately 70–80 days in a humid, organic matter rich environment that is warmed periodically, which makes it ideal for the growth of micro‐organisms (Hiscox, 2014). Therefore, adaptive variation is expected between the different species in terms of the eggshell physical structure to respond to each climatic regime and altitude.

Previous studies on Apteryx have suggested that their water vapor conductance and porosity is low to reduce the risk of desiccation during the long incubation period (Calder, 1979; Silyn‐Roberts, 1983). However, in most burrowing birds, a higher porosity and conductance have been observed because the humidity in a burrow can be closer to 100%, thus reducing the rate of water diffusion from the egg. There is a potential trade‐off between the incubation period and the nest environment. There are other adaptations that could compensate for the need of increased gas exchange concurrently with a need to prevent microbial contamination. For example, a reduction in pore size through mechanical means such as cuticular particles or opercula partially or fully plugging the pores (Board & Perrott, 1979).

Some hypotheses about the function of the characteristics of the Apterygian egg and the eggshell to respond to certain ecological demands have been proposed. Calder (1979) suggested that the increased amount of ovoinhibitors and lysozymes in the albumen of *Apteryx* eggs function as a last line of defense, given that the eggshells are very thin and the incubation period very long, which drastically increases the risk of microbial infection in damp microbe‐attracting burrows. Prinzinger and Dietz (2002) and Maloney (2008) suggested that the slow metabolic and developmental rate allows the egg to withstand long periods of parental absence. However, characteristics of the eggshell such as interspecific variation in eggshell thickness and water vapor conductance could help clarify the nature of these adaptations in *Apteryx*.

*Apteryx* has been included in some studies on water vapor conductance variability in different species as an example of extreme adaptations (Calder, 1979; Tullett, 1984). However, these data have mostly been based on measurements from what was considered *Apteryx australis*, a species now known to comprise three species and be further subdivided into nine (or more) different taxa inhabiting at different altitudes and experiencing different climatic regimes (Weir et al., 2016). Outside New Zealand, samples are usually obtained from birds bred in captivity, which are known to produce smaller eggs than those laid in the wild (Reid, 1981). Some authors have tried to address these questions but unfortunately with very few eggs and eggshells (Silyn‐Roberts, 1983), leaving this matter open to be researched in more depth.

In this study, we examined the eggshell structure of four species of *Apteryx* and contrasted the findings with previous hypotheses regarding the expected structure. Eggshell thickness is known to have a strong positive relationship with adult body mass (Birchard & Deeming, 2009); therefore, we analyzed measurements of eggshell thickness of the four species of Apteryx with adult body mass reported in the literature. Trade‐offs between a long incubation (requiring a decreased water vapor conductance), the burrow nest (which would require a higher water vapor conductance to tolerate the high humidity of the nest), and adaptation to altitude (a reduced conductance to tolerate lower barometric pressure) suggest that each species should have adaptations to their particular habitats. Based on each species distribution, it is expected that species inhabiting higher altitudes, such as Tokoeka, will have lower water vapor conductance than the lower altitude species, Brown Kiwi and Rowi (Carey, 1980; Rahn et al., 1977a, 1977b). Water vapor conductance can be modulated by changing the ratios of pore functional area (Ap) and eggshell thickness (L). We would therefore expect to observe a reduction in pore functional area and/or an increase in eggshell thickness in species of *Apteryx* living at high altitudes. Ambient temperature can also influence water vapor conductance since water diffusivity in air proportionally increases with temperature; therefore, species living in warmer areas are expected to have either thicker eggshells and/or smaller pores. The eggshell thinning process associated with the calcium intake by the developing embryo has not been assessed for Kiwi; here we propose an estimation based on previous measurements of water vapor conductance of infertile eggs and the measured water vapor conductance and thickness of hatched eggs.

## 2 MATERIALS AND METHODS

### 2.1 Eggs

All kiwi species are in a precarious conservation situation mostly because of the ravages of introduced mammalian predators (Germano et al., 2018). Populations are primarily declining due to predation of chicks by stoats (*Mustela erminea*) and adults by ferrets (*Mustela furo*) and unruly dogs (*Canis familiaris*) (Germano et al., 2018). Eggshell measurements were obtained from Operation Nest Egg (ONE), a program for the captive rearing of wild *Apteryx* eggs (Holzapfel et al., 2008). Its purpose is to assist increasing wild population numbers by hatching wild‐laid eggs in captivity and rearing the chicks until they gain enough weight to survive predation by introduced mammal species before release back into the wild (Colbourne et al., 2005). The eggs in this program are generally collected after day 20 of incubation to increase the hatching success through artificial incubation. The eggs used in this study were retrieved after day 35 (range 35 to 60) and thoroughly cleaned upon arrival to the artificial incubation facilities to eliminate mud and debris from the eggshell surface. After captive hatching eggshells were collected by staff members at Rainbow Springs (Rotorua), West Coast Wildlife Centre (Franz Josef), and Paparoa National Park, and stored in sealable plastic bags with an identification code (collection locality and incubating male ID) and kept at room temperature until used in this study.

Eggshells from 30 Brown Kiwi (*A. mantelli*), four Roroa or Great‐spotted Kiwi (*A. haastii*), 25 Rowi (*A. rowi*), and 20 Haast Tokoeka (*A. australis australis*) from the 2013–2017 breeding seasons were used (Table 1).

**TABLE 1:** Information regarding species and their weights (BK = Brown Kiwi, GS = Great‐Spotted Kiwi, R = Rowi, T = Tokoeka), sites of origin of the eggshell samples, and conditions at the sample sites

Sp.	Location	Max. weight (Kg)		Co‐ordinates		DAI (Mean ± *SD*)	EA (Mean ± *SD*)	No. eggs
		Males	Females	Lat	Long			
BK	Oh	2.0	2.8	−37.980	177.028	26 ± 12*	48 ± 12*	3
	Ma			−38.809	176.783			7
	To			−39.159	175.550			12
	Co			−37.054	175.666			8
GS	Pa	2.3	3.0	−42.021	171.360	unknown	unknown	4
R	Ok	2.4	2.5	−43.273	170.176	39 ± 12	35 ± 12	25
T	Ha	2.3	3.0	−44.025	168.214	36 ± 17	38 ± 17	20

Note

DAI = Number of days in artificial incubation, EA = Estimated age at the start of incubation in captivity.

* Average value for all Brown Kiwi samples. Data on weights from Castro and Morris (2011). Locations are Co = Coromandel, To = Tongariro, Oh = Ohope, Ma = Maungataniwha, Pa = Paparoa National Park, Ok = Okarito Forest, Ha = Haast Sanctuary.

**FIGURE 1:**
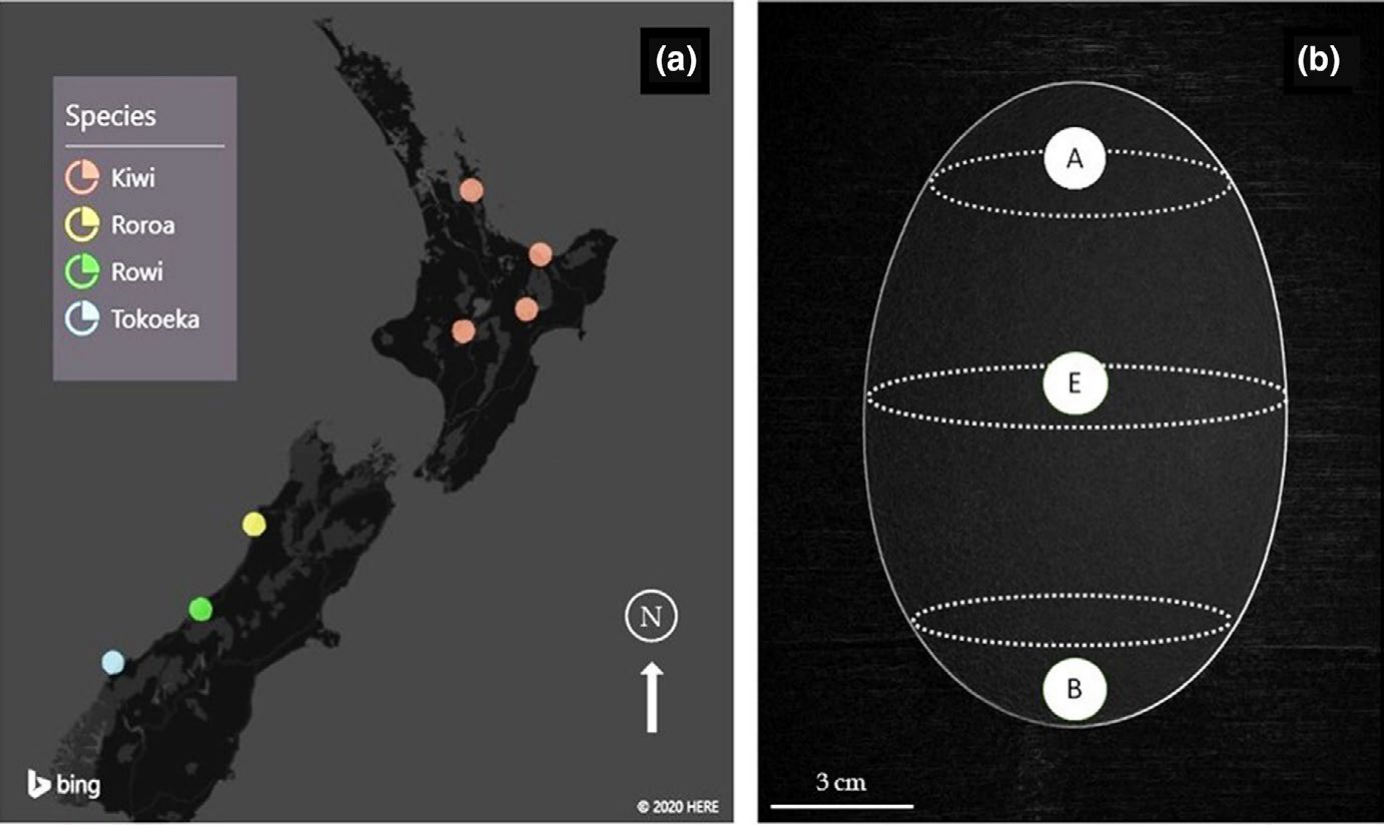
(a) Partial map of New Zealand showing the locations where egg where samples were collected. (b) Modified photograph of a Brown Kiwi egg showing the different regions of the egg where samples were taken from; A = acute or pointy end, E = Equator, and B = blunt end

The eggshells originated from seven locations in the two islands of New Zealand (co‐ordinates in Table 1, Figure 1a). Brown Kiwi is restricted to the North Island, and it generally experiences milder temperatures and rainfall than Rowi and Tokoeka, which are restricted to the Okarito forest and the Haast mountain ranges, respectively, which are both characterized by very cold winters with heavy rain, and occasional snowfall (Table 2). Roroa inhabits highlands at the northern part of the South Island of New Zealand, and it experiences low rainfall and warmer temperatures than the two southernmost species (Germano et al., 2018).

**TABLE 2:** Climatic data are an average of the monthly data from April 2015 to February 2016 obtained from World Weather Online (www.worldweatheronline.com/lang/en‐nz)

Species	Location	IS	Pluviosity (mm)		Temperature (°C)		Pressure (mbar)	
			Mean	*SD*	Mean	*SD*	Mean	*SD*
BK	Co	M	46.03	17.05	15.36	3.23	1,017.35	1.62
	To		39.92	21.13	9.18	4.26	1,016.20	1.40
	Oh		53.44	21.49	11.64	4.11	1,016.73	1.52
	Ma		27.80	18.43	13.27	3.64	1,016.19	1.59
GS	Pa	MF	35.25	21.00	15.27	3.29	1,017.68	1.83
R	Ok	MF	104.35	35.34	4.00	4.75	1,014.01	1.36
T	Ha	MF‐Co.	140.74	55.26	8.91	3.21	1,014.09	1.29

Note

Locations are Co = Coromandel, To = Tongariro, Oh = Ohope, Ma = Maungataniwha, Pa = Paparoa, Ok = Okarito, Ha = Haast. Incubating strategy (IS) represents the involvement of parents in in the incubation process; M = male incubation only, MF = male and female, and MF‐Co = male, female and helpers. Species (BK = Brown Kiwi, GS = Great‐Spotted Kiwi, R = Rowi, T = Tokoeka).

To ensure the climatic comparisons were accurate, only eggshells from eggs that hatched during the breeding season 2015–2016 were used, since these comprised most of the samples. The eggshells were relatively intact except for the section broken by the chick during hatching. All the eggshell fragments were gently rinsed with deionized water and let to air dry for at least 12 hr.

Statistical comparisons used different numbers of eggshells depending on the initial state of the eggshells and the purpose of the comparison. Rahn and Ar (1980) suggested that pore distribution varies according to the latitude of the egg; therefore, for the water vapor conductance comparisons of the three brown *Apteryx* species, samples were taken manually from the equatorial region of each eggshell (Figure 1b) to ensure that measures were comparable across species. This area of the egg was chosen for two reasons: First, because of hatching, the blunt end of most egg samples was destroyed, this being the usual exit point for the chick, and second, for some of the samples the eggshells were somewhat crushed, and the equatorial region was the only identifiable part of the eggshell. However, some of the eggs were intact enough to identify different eggshell regions; in these cases, a fragment from each, the pointed end, the equator, and the blunt end were used to compare the water vapor conductance of the different regions.

### 2.2 Imaging techniques

Eggshell fragments from the equatorial region of the egg were used to assess variation in egg porosity and thickness of each shell layer between each species. The fragments were manually broken and thoroughly washed in deionized water and allowed to air dry. The eggshells were relatively clean as they were cleaned thoroughly during artificial incubation at ONE facilities by gently rubbing them with cotton buds moistened with distilled water.

#### 2.2.1 Micro‐computed tomography

Micro‐CT (Zeiss, MicroXCT‐L, Xradia Inc., Concord, CA, USA) was used to determine the radii of pores and their geometry. Nine eggshell fragments from the equatorial region of the egg belonging to the four species of *Apteryx* were used. The fragments were manually broken into three smaller fragments each, and a full scan of each fragment was used to observe the pore geometry; the resulting fragments had an approximate area of 0.03 cm^2^ (*SD* = 0.001). Images were obtained at 45 Kj, 133 μA, 6 W; a source distance of 20 mm, detector distance at 8 mm, pixel size of 1.9266 μm, objective magnification 10×, exposure time 11 s, and 1,000 projection images through 180° of rotation. The images were analyzed using Xradia mxct software Version 11. Images from the cuticle of all samples were taken to see its physical features.

The diameter of pores that fully transverse the eggshell was measured at three points along the pore, near the external opening, midway, and the internal opening; these measurements were divided by two and averaged to produce a mean pore radius.

#### 2.2.2 Scanning electron microscopy

##### Eggshell thickness

Scanning electron microscopy (SEM) (FEI Quanta 200 ESEM, Eindhoven, Netherlands) was used to measure the palisade thickness, the mammillary thickness, and the total shell thickness from fragments taken from the equatorial region of the eggshell. The samples were rinsed with reverse osmosis water to further remove any particles and allowed to air dry; samples were then mounted on aluminum stubs and gold spluttered with approximately 100 nm of gold in a vacuum (Baltec SCD 050 sputter coater). The images were taken by the Manawatu Microscopy and Imaging Centre (MMIC), using an accelerating voltage of 20 kV and a spot size of 3 to 4 mm. For this technique, eggshells of 23 Brown Kiwi, 4 Roroa, 21 Rowi, and 16 Haast Tokoeka were used.

The cuticle was imaged at 20 μm resolution, and cross‐sectional images and internal side images were produced at 300 μm resolution. The measurements were analyzed using ImageJ 17 free software (Rueden et al., 2017).

##### Pore radii

The average pore radius was measured using SEM images of the external layer (cuticle). Only images that showed opened pores were used (*N* = 87), these images belonged to 28 eggs, and we used ImageJ to take three measurements of diameter per pore. The measurement of diameter was replicated because the pores are not perfectly circular. These measurements were subsequently divided by two to estimate the radius, and the radii were averaged per individual pore. A species average was used for further calculations based on fragments taken from the equator; later averages were used to compare with micro‐CT radius measurements.

#### 2.2.3 Dissecting microscope

**FIGURE 2:**
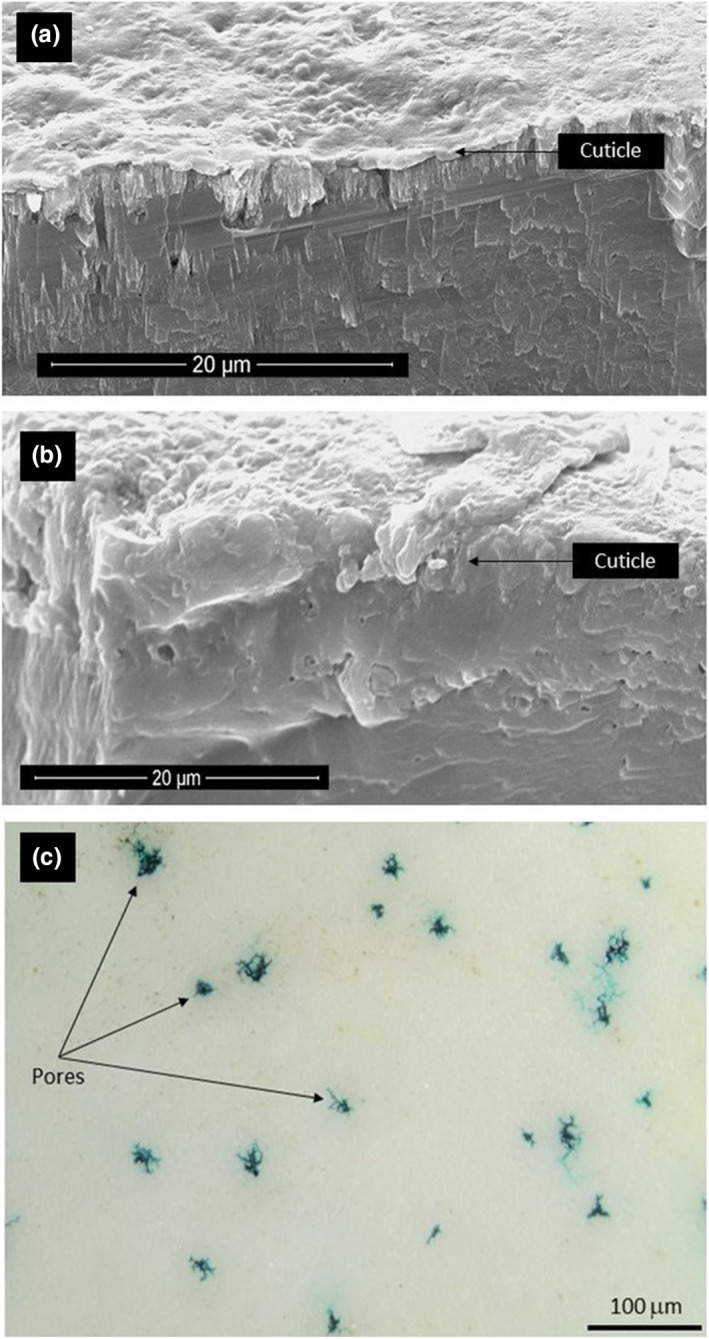
(a) and (b) Cross‐sectional scanning electron microscopy images of a Brown Kiwi eggshell showing the cuticle. (c) External view of the outer surface of a Brown Kiwi eggshell obtained with a dissecting microscope. Staining with malachite green has penetrated through the pores making them visible

The pore density of the three species eggshells was measured under a dissecting microscope (Olympus SZX12). Eggshell fragments (1.11 cm^2^, *SD* = 0.13) from the equatorial region of Brown Kiwi (13), Rowi (12), and Haast Tokoeka (10) were washed in an ultrasonic bath filled with deionized water to remove the plugs from the pores, let air dry, and stained with an alcohol‐based solution of Malachite green (1%). The eggshells were placed on paper towels, and two drops of the dye were applied to the inner eggshell surface. The eggshells were let to set to allow the dye to penetrate the pores without staining the cuticle (Figure 2c).

The eggshells were observed under a stereo microscope (Olympus SZX12) and photographed using an attached camera (Olympus S30), and then, the pores were individually counted using ImageJ to estimate pore density (pores/cm^2^). This means that pore density was estimated considering both the open and the plugged pores.

### 2.3 Water vapor conductance of eggshell fragments

Water vapor conductance was estimated following the methodology reported by Portugal et al. (2010) under controlled conditions, instead of using a whole infertile egg (Ar & Rahn, 1985). Fragments from the equator of the eggshell (*N* = 62) belonging to the three species of Brown Kiwi were glued (Elmer's PVA) to a PCR tube (SSI, 0.5 ml, Cat. No. 1110–02) filled with 200 μl of distilled water, with the inner surface of the shell facing inside the tube, the glue was applied to the outer edge of the shell, so the pores were not tampered. The tubes were placed in PCR trays for easy handling and the trays placed in a desiccator containing 550 g of color‐indicating silica gel to produce a 0% humidity environment, and the desiccator was then placed in a controlled temperature room at 25°C (+0.5). Water loss was measured every 24 hr for three consecutive days by weighing the tubes. Eggshells from commercially produced chicken eggs were included in this experiment as a control to determine if the values obtained in this experiment were congruent with those reported in the literature. The chicken eggs were of an unknown variety, but all were infertile or from very early stages of incubation (blastodisc sometimes observed). Separately, fragments from different eggshell regions, if available, were used in the same way. The fragments were taken from the blunt end (B), the pointed end (A), and the equator (E) of each egg (Figure 1b). The purpose of this was to determine if there were differences between the different eggshell regions and according to species. In many species, the blunt end tends to have a greater water vapor conductance, and therefore, this was expected to be the case for *Apteryx*.

We determined the daily water loss ($\Delta M_{H_{2}O}$) by weight loss and calculated the water vapor conductance as:

(1) $$\Delta G_{H_{2}O}=\Delta M_{H_{2}O}/\Delta P_{H_{2}O}$$

where $\Delta G_{H_{2}O}$ is the water vapor conductance, and $\Delta P_{H_{2}O}$ is the pressure difference at standard conditions (1 atmosphere and 25°C). The air cell pressure and nest environment pressure difference has been calculated for most avian species, including burrow nesters to be 23.77 mg d^−1^ torr^−1^ (Ar et al., 1974). Therefore, we used this value as the water pressure difference ($\Delta P_{H_{2}O}$ ).

### 2.4 Water vapor conductance of eggs during incubation

Eighteen fertile Brown Kiwi eggs were used to determine the water vapor conductance at late stages of incubation. In ONE, the eggs are incubated at a constant temperature of 35.5°C and ~60% humidity. The eggs were weighted at the ONE facilities upon arrival and before hatching, to account for water loss. Using this information, and the number of days in artificial incubation before hatching, we calculated the water vapor conductance of the eggs at late stages of incubation. We used the relative humidity equation to calculate the pressure difference between the interior of the egg and the incubator (considering that the pressure difference is 23.77 Torr in 0% humidity at 25°C).

(2) $$p_{H_{2}O}=\frac{\text{RH}*p_{H_{2}O}^{*}}{100\%}$$

Here, $p_{H_{2}O}$ is the partial pressure of water vapor in the incubator, RH is the relative humidity (60%), and *p_H2O_* is the total pressure of water vapor at 35.5°C.

Water vapor conductance was calculated for late incubation using Equation 1 and then corrected to 25°C using the conversion factor reported by (Bucher, 1983; Paganelli et al., 1978):

(3) $$\;{\left(\frac{298}{T_{a}+273}\right)}^{0.5}$$

where 298 is equivalent to 25°C, and Ta is the incubation temperature (35.5°C). Since the eggs are collected approximately at the midpoint of incubation (35 days), the fresh mass of the egg was estimated using the equation reported by (Reid, 1971) specific for Brown Kiwi:

(4) $$0.565\;ab^{2}$$

where *a* is the length of the egg (or the distance between the pointed end and the blunt end) and *b* is the width of the egg.

To compare our results with allometric predictions, we calculated the water vapor conductance using the fresh egg mass based on the equation reported by Ar et al. (1974):

(5) $$G_{H_{2}O}=0.432*W^{0.780}$$

where *W* is the fresh weight of the egg.

Ideally to examine the thinning of the eggshells, the eggshell thickness of fresh versus hatched eggs should be compared, but we did not have access to fresh eggs from any of the areas where we obtained eggs as these were wild eggs incubated by wild parents. Therefore to estimate the eggshell thinning through incubation, we assumed the value of water vapor conductance reported by Calder (1979) as the initial conductance and the values calculated in this study as final conductance and used Equation 1 to estimate the water loss in each stage by rearranging the equation as follows:

$$\Delta M_{H_{2}O}=\Delta G_{H_{2}O}*\Delta P_{H_{2}O}$$

And using the equation reported by Ar et al. (1974) to calculate porosity, or the relationship between pore functional area (*Ap*) and eggshell thickness (*L*):

$$M_{H_{2}O}=c*D_{H_{2}O}*\frac{\mathrm{Ap}}{L}*\Delta P_{H_{2}O}$$

And resolving for *Ap/L*:

(6) $$\frac{\mathrm{Ap}}{L}=\frac{M_{H_{2}O}}{c*D_{H_{2}O}*\Delta P_{H_{2}O}}$$

Finally, assuming that *Ap* remains constant throughout incubation we calculated the initial thickness (L_i_) using the average thickness measured using SEM as final thickness (L_f_).

### 2.5 Statistical analysis

A discriminant analysis was used to determine the degree of association of each eggshell sample with the donor species to determine the degree of differentiation between the four species of *Apteryx*.

A linear regression was used to test the allometric relationship between eggshell thickness and adult body mass; in this case, we used the adult weights reported by Ramstad and Dunning (2020) for both females and males, as in all species male incubate and in Brown Kiwi only the male incubates. The eggshell thickness was squared (L^2^), and both variables were log transformed, following the methods of Birchard and Deeming (2009).

One‐way ANOVAs were used to see if there were any differences between the eggshell thickness, pore radii, and pore density between the three species of brown colored Kiwi. A pairwise comparison was done between the SEM measured pore radii and the averaged pore measurements obtained with micro‐CT.

A spearman rank correlation was performed to test associations between eggshell thickness, pore density, and pore radius with environmental variables: temperature, barometric pressure, and pluviosity. For this, we used weather information from the area the egg was collected; the average monthly temperature, pluviosity, and barometric pressure from April 2015 to February 2016 was obtained from worldweatheronline.com.

A one‐way ANOVA was performed using the samples from Brown kiwi, Rowi, and Haast Tokoeka to assess potential interspecific differences in total eggshell thickness and cuticular thickness (the outermost mineral layer differentiated from the smooth vesiculated crystalline layer, Figure 2a).

Roroa was excluded from further statistical analysis due to insufficient sample size.

An ANOVA was used to test if there were differences in water vapor conductance between the species, and a nested linear model was used to test differences between eggshell regions within each species. In this case, the fragments (E, A, and B) were nested within species. All the ANOVAs performed in this study were followed by a Tukey *post hoc* test to assert which species differed from the others. A repeated measures ANOVA was used to determine the difference in water vapor conductance of three different regions of the eggshell of Brown kiwi, Rowi, and Haast Tokoeka and to determine the extent of the interaction of the species and the region of the eggshell.

A paired *t* test was performed to compare the water vapor conductance of whole artificially incubated Brown Kiwi eggs and the water vapor conductance calculated from Equation 5. using the calculated fresh mass of the egg. All statistical analysis was conducted in MiniTabTM statistical software (version 18).

## 3 RESULTS

### 3.1 The different layers of the Apteryx eggshell

**FIGURE 3:**
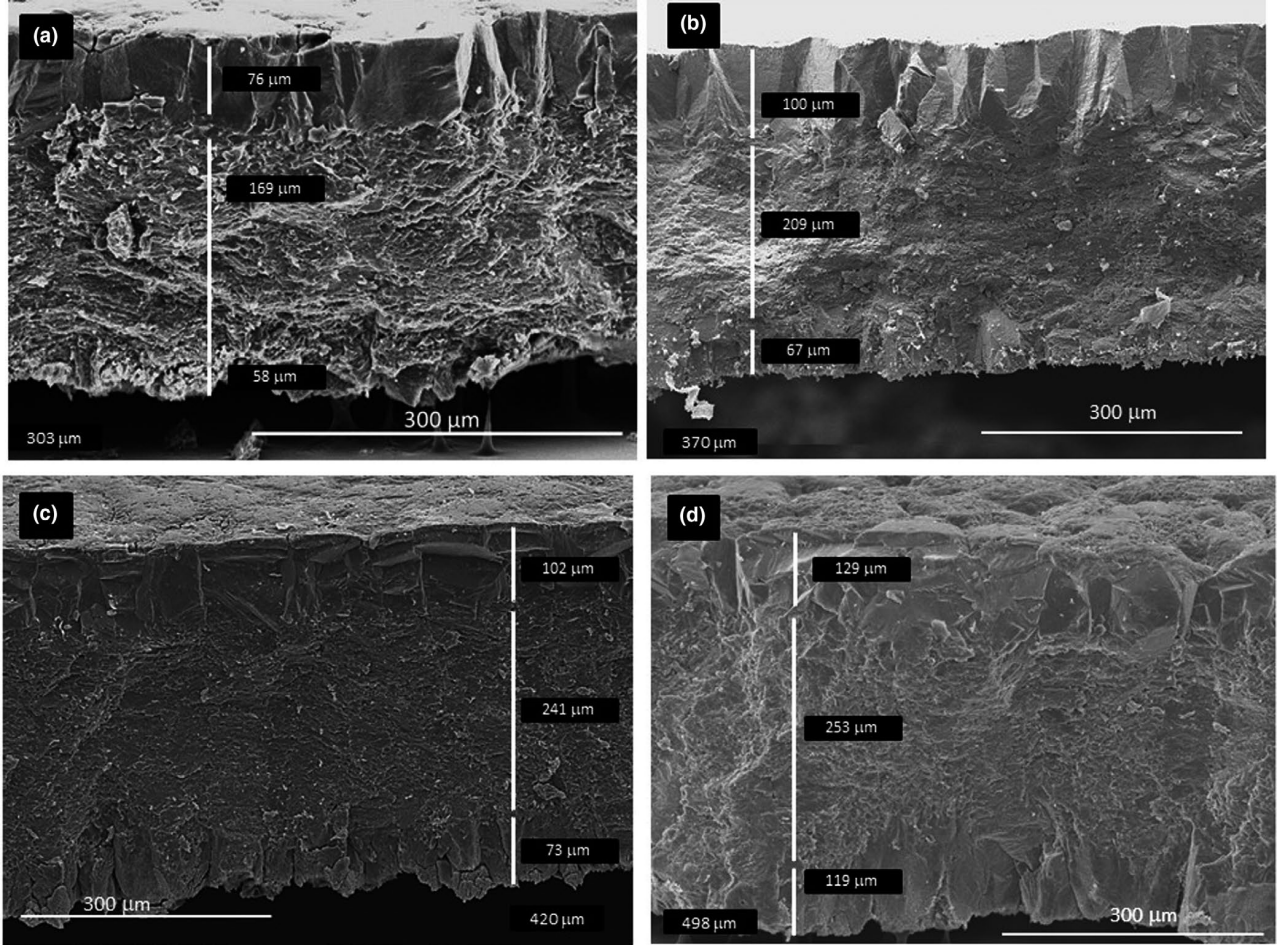
Cross‐section of (a) Brown Kiwi (b) Roroa, (c) Rowi, and (d) Haast Tokoeka eggshells obtained using scanning electron microscopy (SEM). Here, the constituent layers are indicated with the vertical bar and their measurements. C = cuticle, Cr = crystalline, P = Palisade, and M = mammillae. Total thickness is indicated at the bottom of each picture

*Apteryx* eggshells showed a clear demarcation between the four constituent layers (Figure 3). With exception of the mammillary layer (ANOVA, *F* = 1.85, *p* = .150; *n* = 59), the constituent layers of the eggshell were significantly different between species. The proportion of cuticle from the total eggshell thickness (ANOVA, *F* = 8.85, *p* < .001; *n* = 59) and the proportional thickness of the crystalline and palisade layers (*F* = 6.76, *p* = .001; *n* = 59) varied significantly between the four *Apteryx* species (Table 3, Figure 3).

**TABLE 3:** Summary of the eggshell thickness and eggshell cuticle thickness obtained by scanning electron microscopy of the eggshell cross‐section (*N* = 64)

Species	*N*	Total Thickness (m)		Cuticle Thickness (m)	Cuticle proportion	Crystalline proportion	Palisade proportion	Mammillary layer proportion
Brown Kiwi	23	304.77^a^		4.07^a^	0.014	0.21	0.58	0.16
	*SD*	8.55			0.005	0.04	0.07	0.03
Rowi	21	368.95^b^	10.61	3.04^b^	0.008	0.20	0.59	0.18
	*SD*	12.50			0.003	0.03	0.07	0.05
Tokoeka	16	383.96^b^	12.56	3.03^b^	0.009	0.18	0.63	0.20
	*SD*	11.76			0.004	0.03	0.06	0.06
		*F* value 15.50	*p* value > .0001	*F* value 6.16	*p* value .004			
Roroa	4	372.20	2.12	3.30	0.009	0.25	0.51	0.20
		29.44			0.005	0.02	0.05	0.05

Note

Roroa were not included in the analysis due to the very small sample size. The proportional thickness of the four constituent layers of the calcified eggshell is reported. Values represent means and standard deviation. Superscripts next to the species name represent the Tukey post hoc test indicating which are the significantly different species.

**FIGURE 4:**
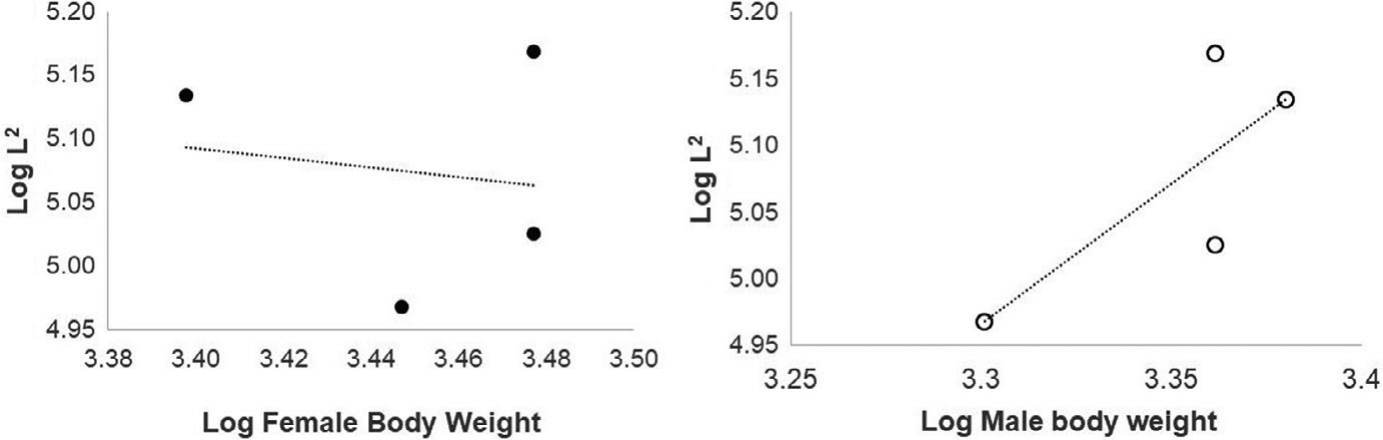
Linear regression between eggshell thickness (L2) and female body weight (full circles) (*R* ^2^ = 0.025, *p* = .85) and male body weight (empty circles) (*R* ^2^ = 0.61, *p* = .22)

Female adult body weight showed a weak negative relationship with eggshell thickness (*R* = −0.15) while male body weight showed a strong positive relationship (*R* = 0.78); however, neither of these relationships were significant (Figure 4).

### 3.2 Interspecific comparison

#### 3.2.1 Discriminant analysis

Using simultaneously the thickness of the eggshell, the mammillary density and area, and the thickness of the different constituent layers, it was possible to associate a particular eggshell to its right species in 78% of the cases (Brown Kiwi = 76.5%, Roroa = 75%, Rowi = 78.9%, Tokoeka = 80%).

#### 3.2.2 The cuticle (or external layer)

All *Apteryx* species presented thin cuticles with a wax‐like appearance, which in contrast to the smooth sponge‐like texture of the crystalline layer made it easy to identify (Figure 2a). Seen from outside the cuticle was smooth and unpigmented, pores seemed to radiate from underneath the cuticle (Figure 2b).

**FIGURE 5:**
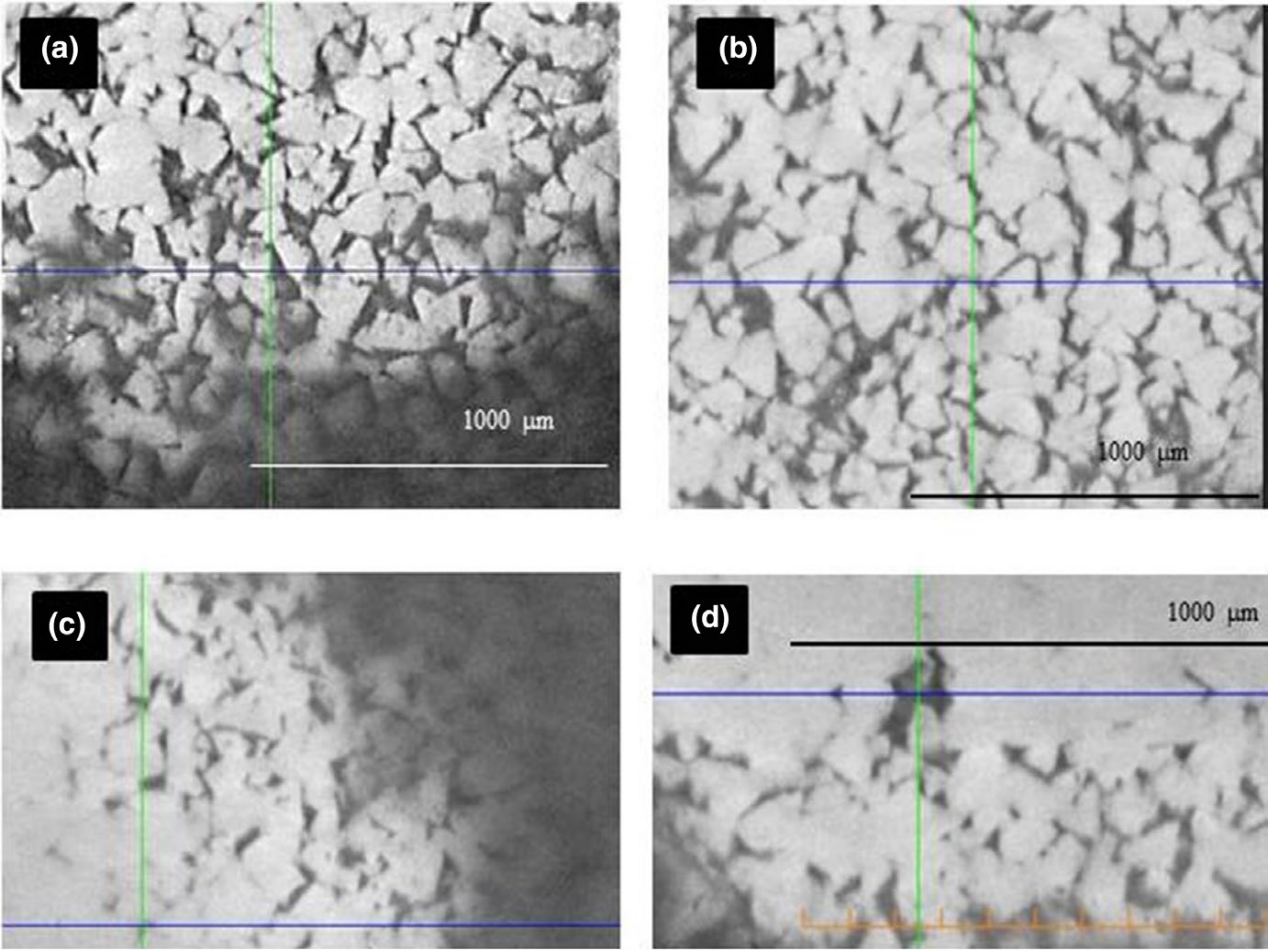
Comparison of the view of the cuticle of (a) Brown Kiwi, (b) Roroa, (c) Rowi, (d) Haast Tokoeka. Using micro‐computed tomography (micro‐CT), it is possible to see the individual triangular crystals that form the cuticle and that are present in all Apteryx species

The cuticle thickness decreased significantly with geographical latitude, being thicker in Brown Kiwi and thinnest in Rowi and Tokoeka; the significance was due to differences between Brown Kiwi and Rowi (Table 3). Under the micro‐CT, the crystalline structure of the cuticle became apparent, and it was possible to discern the individual crystals that formed it (Figure 5).

#### 3.2.3 The pores

**FIGURE 6:**
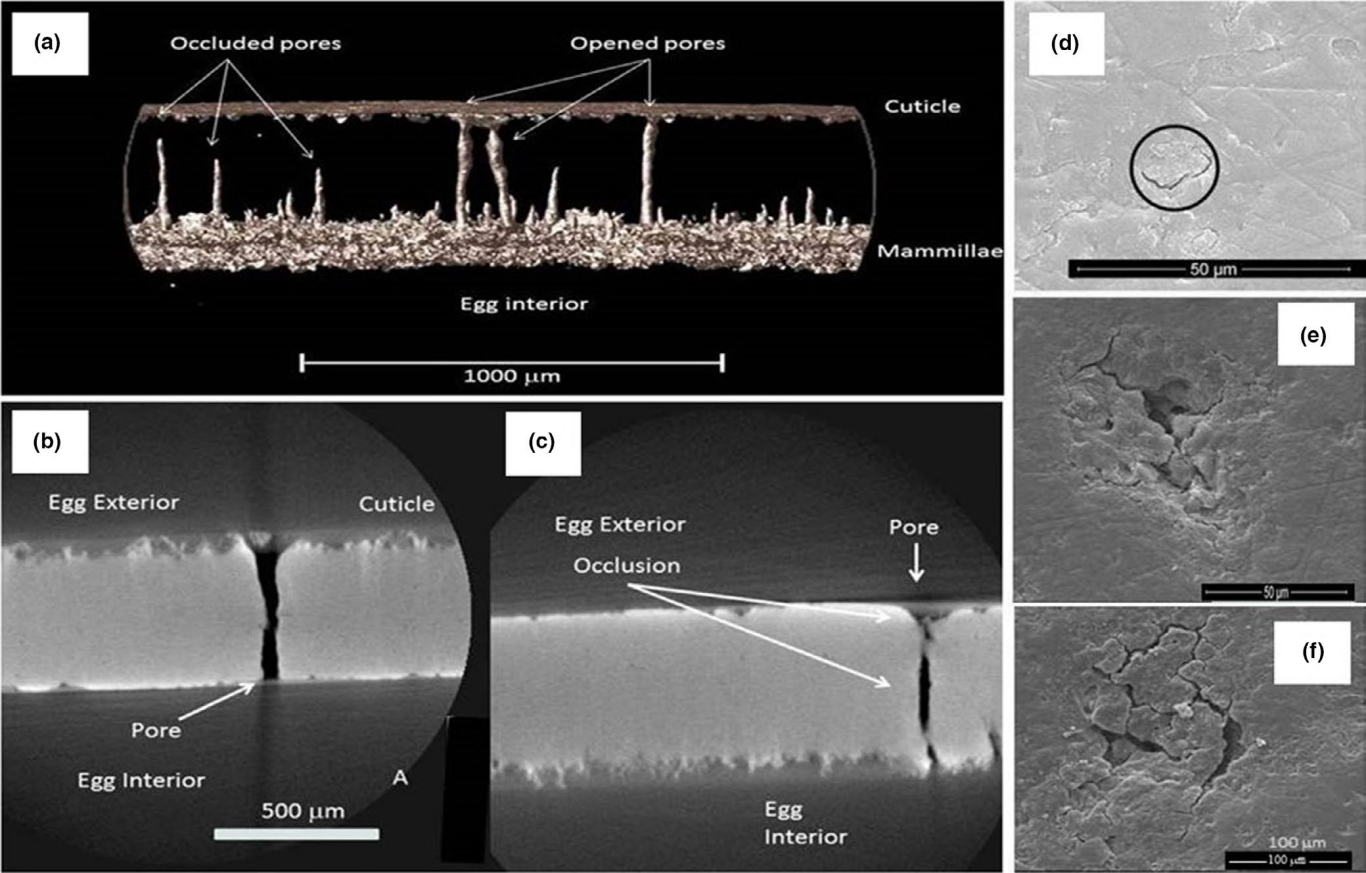
Pores of an Apteryx eggshells visualized by micro‐CT (a–c) and scanning electron microscopy (d–f). (a) transversal view of a Brown Kiwi eggshell, the spaces have been inverted and the pores are shown as solid columns. (b) Brown Kiwi eggshell showing an open pore. (c) The same eggshell showing another pore, in this case occluded with mineral material. External views of capped pores on (d) Brown Kiwi (e) Rowi and (f) Tokoeka

Some pores were hard to identify in the external images of the eggshells because a “cap” made from the same mineral as the cuticle (Figure 6) covered them. *Apteryx* eggshells had cylindrical (or funnel shaped) pores that usually crossed the thickness of the eggshell occasionally being sinuous (Figure 6a). In each fragment, very few pores reached the cuticle surface of the eggshell, with most reaching only half or less of the total thickness of the eggshell and many smaller pores that barely went beyond the mammillary layer, the latter being most likely points where calcium was taken for embryo development thus increasing the gap between mammillary nodes (Figure 6a).

**FIGURE 7:**
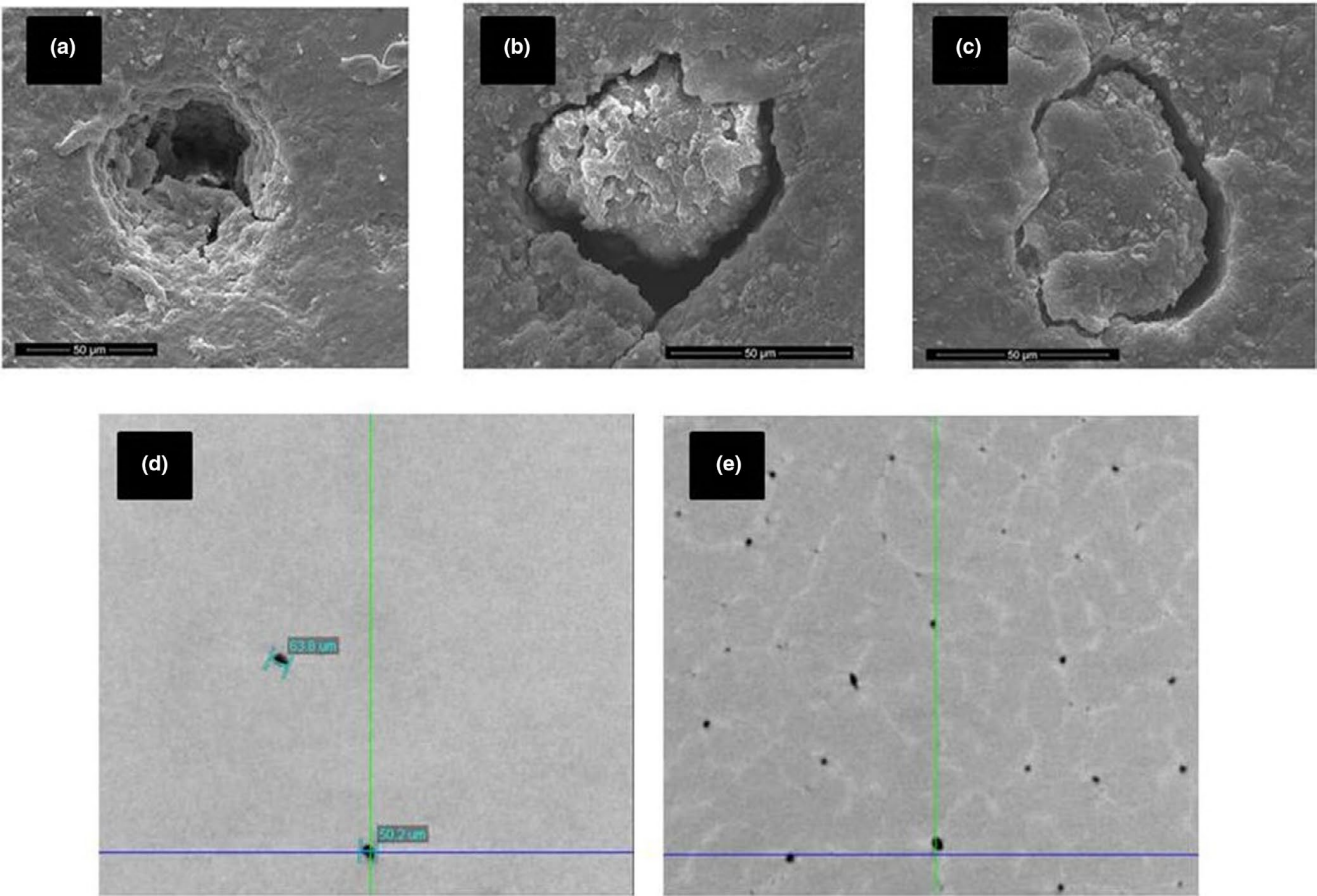
Scanning electron microscopy (a‐c) and micro‐CT (d‐e) images of the plugs inside the Apteryx eggshell. (a) an open pore (b) and (c) plugged pores. (d) View from the exterior toward the inside of two pores near the surface of a Brown Kiwi eggshell (e) same fragment, this time halfway through the eggshell showing more pores and the outline of the calcium units from the mammillae as white lines

The observations made using the micro‐CT were confirmed by SEM images of the exterior of the eggshells, where in some cases caps were seen covering the pores and in others mineral occlusions were observable (Figure 7). These caps and occlusions were observed in all four species and in all eggs.

Accordingly, fewer pores were visible at the cuticle side of the egg and many more from the inside. Since the micro‐CT is an X‐ray technique, it is possible to see at different “depths” through the eggshell. When observed near the cuticle, few pores were discernible but when looking midway between the cuticle and the mammillae more pores become visible, confirming what was seen in the cross sections (Figure 7d,e). The number of incomplete pores was always greater than that of complete pores (67% of all pores, *SD* = 17.6); for this comparison, we counted only the incomplete pores that reached halfway the eggshell thickness, to differentiate from those that could be caused by the embryogenic eggshell erosion. There is no significant difference between species in the proportion of incomplete to complete pores (*F* = 1.65, *df* = 3, *p* = .29).

#### 3.2.4 Pore radius and pore density

Pore size measured as average pore radius was different between the southern species and Brown Kiwi, the latter having smaller pores; pore density in contrast was higher in Brown Kiwi and lowest in Tokoeka (Table 4). The measurements taken using micro‐CT were in accordance with those obtained with SEM, except for Tokoeka. In Tokoeka, the pores seemed to thin midway, having a larger outside opening and a broader base. Using both techniques revealed significant differences between in average pore radius between species. In both cases, Rowi was significantly different to the other species (Table 4). A comparison between the observations using SEM and micro‐CT was significantly different (T = 2.49, *p* = .02).

**TABLE 4:** Summary of the pore density, and average pore radius

Sp.	*N*	Pore density (pores/cm^2^)			Pore radius* (μm)		*N*	Pore radius^⁑^ (μm)		
		Mean	MSE	PR	Mean	MSE		mean	*SE*	PR
BK	13	51.3^a^	3.12	40.5 ± 1.9^†^	22.90^a^	2.81	3	18.43^a^	0.40	11.23⁂±3.58^†^
R	12	45.7^ab^	3.25		33.04^b^	2.69	2	32.79^b^	0.43	24.5†
T	10	36.1^b^	3.56		31.80^ab^	3.81	2	18.27^a^	1.75	
*F* value	5.19	3.74		9.98						
*p* value	.012	.04		.015						
GS							2	17.99^a^	4.87	

Note

Superscripts indicate the statistical differences found using Tukey's test, values within columns with different superscript are significantly different from each other (*p* ≤ .05). *Data obtained by measuring SEM images. ^⁑^Data obtained by averaging three measurements along individual pores visualized using micro‐CT. ⁂Calculated from reported individual pore area. PR = previously reported data (Silyn‐Roberts, 1983 ^†^; Toien et al., 1988 ^†^).

#### 3.2.5 Eggshell thickness

Eggshell thickness varied with species and seemed to follow a latitudinal pattern with thinner eggshells in the northernmost species and thicker eggshells in the southernmost species (Table 3); Roroa's eggshell thickness is indicated in the table for comparison.

#### 3.2.6 Water vapor conductance of eggshell fragments

**FIGURE 8:**
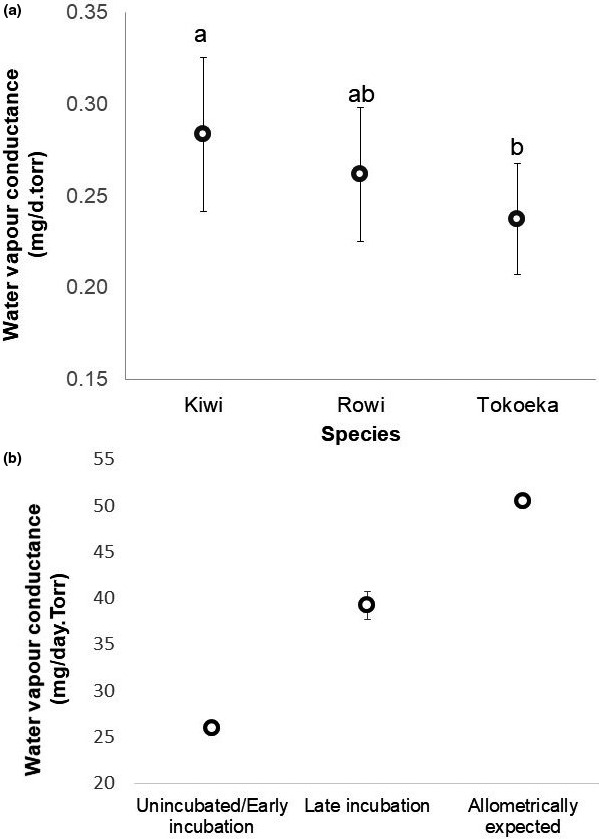
(a) Mean values of water vapor conductance of eggshell fragments for three species of Apteryx. Kiwi *N* = 27, Rowi *N* = 21 and Haast Tokoeka *N* = 14. Means with different letters have significant statistical differences between them. The bars represent the standard error. (b) Comparison of the water vapor conductance of Brown Kiwi reported for an infertile egg (Calder, 1978), the water vapor conductance measured in this study for 18 eggs in the last 26 days of incubation, and the expected conductance calculated from fresh weight

The three species of Brown Kiwi differed significantly in the water vapor conductance of their eggshells (Figure 8a), with Brown Kiwi having a significantly higher water vapor conductance when compared to Tokoeka (*F* = 3.87, *p* = .001), and Rowi being significantly different to Tokoeka (*F* = 2.06, *p* = .05). Rowi did not differ significantly from Brown Kiwi (*F* = 1.91, *p* = .062). Eggshell fragments from the blunt end generally had a greater water vapor conductance than the pointed end and the equator, which showed no significant differences (Table 5). There was no interaction between species and fragment.

**TABLE 5:** Repeated measures analysis of the three different eggshell regions in Brown Kiwi, Rowi, and Tokoeka

Species			Fragment		
	Least Square Mean	*SE*		Least Square Mean	*SE*
Kiwi^a^	0.24	0.013	A^a^	0.23	0.013
Rowi^b^	0.28	0.015	E^a^	0.23	0.013
Tokoeka^a^	0.23	0.012	B^b^	0.28	0.013
*F* value	3.15		*F* value	3.52	
*p* value	0.05		*p* value	0.02	
Interaction Species *Fragment					
*F* value	2.06				
*p* value	0.11				

Note

This is a comparison of water vapor conductance between eggshell fragments from the same egg; pointed end (A), equator (E), and blunt end (B). The superscript indicates significant differences between regions with different letters.

#### 3.2.7 Climatic variables

All the climatic variables showed moderate to low correlations with eggshell physical parameters (Table 6). Pluviosity showed significant relationships with all the eggshell physical characteristics, while pore density was not correlated with temperature or barometric pressure. Thickness was moderately associated with pluviosity, temperature, and barometric pressure, and all these associations were significant.

**TABLE 6:** Spearman rank correlations between environmental variables and eggshell physical characteristics of Brown Kiwi, Rowi, and Tokoeka

Eggshell character	*N*	Pluviosity		Temperature		Pressure	
		Spearman coefficient	*p* value	Spearman coefficient	*p* value	Spearman coefficient	*p* value
Eggshell thickness	42	**0.59**	**<.001**	**−0.51**	**<.001**	**−0.42**	**.006**
Pore density	37	**−0.51**	**.001**	0.11	.51	0.10	.57
Pore radius	29	**−0.36**	**.05**	**−0.43**	**.02**	**−0.40**	**.03**

Note

Statistically significant associations are in bold.

Samples are taken from the equator of the eggshell.

#### 3.2.8 Water vapor conductance of eggs during incubation

The water vapor conductance of whole eggs was significantly different to that calculated from allometric equations using the egg's fresh mass (*df* = 17, T = −6.96, *p* > .001) (Figure 8b).

We used the values reported in the literature to estimate the initial water vapor conductance and the water loss at that stage (Table 7). Calder (1978) used infertile eggs and the methodology employed by Rahn and Ar (1974).

**TABLE 7:** Calculated eggshell thinning based on the water vapor conductance of an infertile egg (Calder, 1978) and the water vapor conductance of artificially incubated eggs during the last 26 days of incubation (*N* = 18)

	Early incubation/Infertile	Late incubation/Hatched
Water vapor conductance	26.0 mg day^−1^ torr^−1^	39.24 mg day^−1^ torr^−1^
Water loss	618.02 mg day^−1^	932.73 mg day^−1^
Ap/L	1.11 cm	1.67 cm
L	**0.045 cm**	0.03 cm (*SD* = 0.003)
Eggshell thinning	0.015 cm	

Bold value indicates the calculated eggshell thickness.

#### 3.2.9 Eggshell thinning

The eggshell was calculated to reduce in thickness about 32.5% from the beginning to the end of the incubation process (Table 7). The calculated initial thickness was consistent with what has been reported in other studies (Calder, 1979; Silyn‐Roberts, 1983).

## 4 DISCUSSION

In this study, we identified species‐specific distinctions in eggshell physical characteristics among members of *Apteryx*. Whether these differences are due principally to phylogeny or ecology is debatable. Some of the species have suffered severe contraction to their ranges, and in some species, all individuals left remain in a single population (i.e., Rowi and Roroa).

Eggshell thickness is known to be proportional to the body mass of the laying bird (Birchard & Deeming, 2009; Tullett, 1978). However, it is worth to notice that the egg mass is very high in relation to body mass, being 23.6 and 14.6% for *A. owenii* and *A. australis* (possibly *A. mantelli*) (Dyke & Kaiser, 2010). The eggshell thickness in of *Apteryx* did not seem to scale proportionally with female body weight, but more likely with male body weight; however, none of these associations was significant, suggesting that the differences in water vapor conductance between species are probably due to adaptations to the environmental conditions each species inhabits, such as barometric pressure, nest humidity, and temperature rather than the size of the adult birds. The incubating system is also very likely to drive the evolution of eggshell characteristics (Birchard & Deeming, 2009), and this possibly explains the better fit with male body weight; however, in three of the four studied species both male and female incubate; therefore, a method to account for the contribution of both parents is needed and to account even by the weight of helpers as it might be the case of Tokoeka. These relationships should also be revised using eggshells from infertile eggs, because by using eggshells from hatched eggs the calcium utilized by the embryo is not accounted for, and it is not known if all the *Apteryx* species utilize calcium at the same rate.

### 4.1 The cuticle and calcified eggshell

The cuticle of *Apteryx* was found to consist of a very thin mineral layer (3–6 μm), with a waxy appearance and water repellent properties. The proportional thickness of the cuticle in respect to total eggshell thickness was very low in comparison with other avian species, being between 0.8% to 1.4% for *Apteryx*, 5.2% for the Mallefowl (Booth & Seymour, 1987), 8.4% for the Adelie Penguine (Thompson & Goldie, 1990), 18.9% for the Greater Flamingo (*Phoenicopterus ruber*), 14.7 in the White Pelican (*Pelecanus onocrotalus*), 7.5% in the Humboldt Penguin (*Spheniscus* humboldti), and 5.6% in the Japanese Quail (*Coturnix japonica*) (Kusuda et al., 2011). The cuticle of *Apteryx* was proportionally thicker than the cuticle of the Domestic Chicken (*Gallus gallus*) being 0.3% (Kusuda et al., 2011). The cuticle was composed of triangular nano‐particles (approximately 100 nm on their side) that formed aggregations that occluded some of the eggshell's pores. Here we present evidence that this characteristic is common to four species of *Apteryx*.

The triangular particles in the cuticle of *Apteryx* have not been reported for any extant bird species investigated so far, but similar ones were observed in the fossil eggshells of *Trigonoolithus amoei* a theropod from the lower cretaceous period found in La Cantalera, Spain. This theropod also presented unbranched pores (Moreno‐Azanza, 2013). The nesting behavior for this dinosaur has not been reported; however, it has been suggested that many dinosaurs might have buried their eggs (Tanaka et al., 2015), and there is evidence of a nesting theropod (*Oviraptor philoceratos*) which might have similar nesting behaviors to modern ratites (Norell et al., 1994); it could be possible to suggest that these triangular particles play some role related to incubation of buried or partially buried eggs. Brown kiwi is known to partially bury its eggs during incubation (Colbourne, 2002). These triangular particles may prevent dirt blocking pores and/or act as a barrier to microbial contamination.

The cuticle and calcified eggshell mediate the interaction between the developing embryo and the external environment. As such, they are the first barriers against pathogens and allow gas and water vapor conductance (D’Alba et al., 2017). Features of the cuticle such as plugs and caps have been associated with nesting in humid environments (D’Alba et al., 2016). Board (1981) mentioned plugs on the eggshell of *Apteryx* and described them being similar to those found on Tinamou, “spheres and fibers both rich in sulfur”; however, the plugs we found seem more similar to those described for the *Rhea americana* on the same study, “irregularly shaped crystalline material”; it is suggested that these plugs serve to avoid water lodging, and depending of the plug shape, it could even function as a valve, in all cases allowing sufficient gas exchange.

*Apteryx* species breed during the austral winter, which is characterized by frequent and heavy precipitation (Leathwick et al., 2002); besides the risk of nest flooding, parents are likely to return to the nest with wet plumage increasing the moisture content of the nests and the possibility of wetting the eggs. We found that pluviosity was significantly and positively associated with eggshell thickness; in contrast, a significant but negative relationship was found between pore density and pore radius with pluviosity, suggesting that these could be adaptations to such wet conditions during incubation by reducing the number and size of pores while increasing the eggshell thickness (length of pores).

Pore radius differed significantly between measurements made with SEM and micro‐CT, this is likely due to the small sample size used in the micro‐CT but also because measurements using SEM only account for the diameter of the external opening, while micro‐CT allows to take measurements in different parts of the pore; so far micro‐CT is the most reliable method to obtain clear measurements of pore properties.

We also found that many pores did not go all the way through the eggshell, a similar observation was mentioned by Silyn‐Roberts (1983). Nevertheless, the function of these types of pores is still not clear. Similar pores that do not transverse the eggshells have been observed in the eggs of Ostriches, another member of the monophyletic group known as Ratites (Maina, 2017; Willoughby et al., 2016). We suggest that future studies should examine the possible role of these partial pores on the strength of the eggshell as well as how these partial pores affect water vapor conductance.

The egg is incubated in a humid environment with frequent presence of liquid water. In this environment, the egg would require adaptations to increase water loss, which would become more important as the incubation progresses; *Apteryx* has a very thin eggshell (27% less than allometrically expected) which increases water loss. Conversely, capped pores reduce water vapor conductance. It seems possible that *Apteryx* increases water vapor conductance in a similar way to Malleefowl by decreasing eggshell thickness, but if the occluded pores open as the incubation process progresses through cuticle abrasion is still not known, but our findings suggest that this is possible (see section: Water vapor conductance of egg during incubation below). Since *Apteryx* does not possess a hatching tooth nor seems to complement its diet with grit or shells during the breeding season and medullary bone as calcium storage has not been confirmed in these species (Dennison & Kooyman, 1991), it could be possible that it has kept a thin eggshell to reduce the needed amount of calcium to produce the eggshell, while increasing the water loss in a humid environment and facilitating the hatching process. Further embryogenic eggshell thinning would increase water vapor conductance as needed.

Water vapor conductance has been observed to decrease with altitude in other avian species, which is generally achieved by a reduction in pore area (Carey, 1980). However, Rahn et al. (1977a, 1977b) indicate that reduction in water vapor conductance can be achieved by changing the ratio of *Ap/L*, meaning that a thicker eggshell (*L*) could also reduce the overall water vapor conductance. In this study, we found that the eggshell thickness of *Apteryx* shows a moderate negative correlation with barometric pressure, meaning that unlike other species the eggshell of *Apteryx* is thinner at lower altitudes and thicker at higher altitudes. Pore radius also showed a moderate negative correlation with barometric pressure, indicating that pore area actually increases with altitude, unlike other studied species (Carey, 1980; Rahn et al., 1977a, 1977b; Sotherland et al., 1980), this happens in the clade of *Apteryx* with brown plumage. Roroa belongs to the clade of spotted *Apteryx* and has the lowest pore area of all the studied species, and it is also the species that inhabits greater altitudes; future studies should compare the water vapor conductance between the two species of the “Spotted” clade, especially since the Little Spotted Kiwi (*A. oweni*) inhabits low elevations, and also to further test the allometric relationship between eggshell thickness and adult body weight.

Water vapor conductance measured on eggshell fragments confirmed these observations being significantly higher for Brown Kiwi when compared to Tokoeka. Tokoeka inhabits generally higher altitudes than Brown Kiwi, and interestingly being bigger in terms of body mass (Ramstad & Dunning, 2020).

Overall, *Apteryx* seems to reduce water vapor conductance as a response to high altitudes; it is likely that there are other factors that favored a thicker eggshell in Tokoeka instead of a more porous one, but possibly obtaining the same result. The water vapor conductance of Roroa, however, was not measured in this study, and it is being assumed to be less due to the reduced pore radius; however, the eggshell is thinner than that of Tokoeka. The exact altitude where the eggs used in this study were laid was unavailable; it is necessary that future studies look at the water vapor conductance at different altitudes to clarify the effect of altitude on eggshell characteristics for this group.

Ambient temperature showed a similar trend with both eggshell thickness and pore radius. In this case, warmer temperatures are expected to increase the evaporative water loss; therefore, the correlation should be positive. However, this was not the case, Brown Kiwi inhabiting warmer climates had a thinner eggshell than Tokoeka and Rowi inhabiting colder climates and experiencing occasional snowfall. This is possibly due to differences in the incubation behavior between these species, in Rowi and Tokoeka both male and female incubate, and the eggs are not left unattended during incubation, conversely only the Brown Kiwi male incubates, and the eggs are left unattended every night while the parent is foraging, usually cooling down to ambient temperature (Colbourne, 2002). The constant presence of a parent in the nest means that temperature is maintained constant and that humidity could be kept generally higher due to respiration and condensation from the incubating parent, while Brown Kiwi eggs would be exposed to more abrupt changes both in temperature and nest humidity. The high altitude Roroa also present biparental incubation but short periods of egg unattendance have been observed (McLennan & McCann, 1991).

In the case of thickness, also differences in incubation behavior could explain this adaptation, as having multiple birds in a nest could increase the risk of breakage, eggshells were generally thicker in the species that present biparental incubation, thus resorting to an increased pore area to compensate for the reduction in water vapor conductance.

The water vapor conductance we measured using eggshell fragments was in accordance to that measured for ground burrowing species reported by (Portugal et al., 2014). The different eggshell regions showed different water vapor conductance indicating a difference either in thickness or pore density. In some eggs, it was possible to observe a cluster of pores concentrated in the most apical extreme of the blunt end; however, since the eggshells used in the study came from hatched individuals in most cases this particular region had fractures and a neighboring fragment was used, possibly underestimating the actual number of pores of that region; nevertheless, it has been noted that the air cell of *Apteryx* is off center (Rowe, 1978). Significant differences were seen between the blunt end and the pointed end for Brown Kiwi and Rowi in water vapor conductance; it is possible that this is also the case for Haast Tokoeka but because of the difficulty of sampling this was not observed.

Calder (1978) showed that the eggshell porosity of Brown Kiwi was 60% of the predicted value by Ar et al., (1974) equation for whole eggs. However, we found that pore density and pore radius were greater than previously measured on our eggshell fragments, indicating that porosity should be higher, this in conjunction with plugged pores could explain why this was found in the past.

### 4.2 Water vapor conductance of egg during incubation

The water vapor conductance of the whole eggs of *A. mantelli* measured in this study was 58% higher as previously reported at 39.24 mg/day torr (*SD* 1.47) compared to 26.00 and 23.71 mg/day torr reported by Calder (1978) and Silyn‐Roberts (1983), respectively. Given that we used eggs that had been incubated up to day 35, and Calder and Silyn‐Roberts used fresh eggs, it seems like the water vapor conductance doubles at about the midpoint of incubation. This is consistent with what was measured by Prinzinger and Dietz (2002), showing that the oxygen consumption increases drastically around this time.

The main way for this increase on water vapor conductance to occur would be through the embryogenic eggshell thinning. Precocial species are known to utilize the most calcium as they are very well developed by the time of hatching (Orłowski & Hałupka, 2015), and in this study, we estimated that the reduction in thickness of the eggshell from laying to hatching is 32.5% of that of the fresh egg thickness. The water loss rate of kiwi is lower than most birds, being about 12.5% (Colbourne, 2002), while for most birds is approximately 18% (Ar et al., 1974), interestingly about 40% of the water loss occurs in the last 26 days of incubation, further indicating a drastic increase in conductance.

Mechanisms to increase water vapor conductance in response to environmental conditions during incubation have been observed in other avian species; Adelie Penguin's eggs (*Pygoscelis adeliae)* have an organic cuticle that plugs the pores reducing the water vapor conductance in early stages of incubation to cope with the extreme aridity of the Antarctic; the abrasion of this cuticle during incubation increases the water vapor conductance to what is expected allometrically for the eggs (Thompson & Goldie, 1990). The Mallefowl (*Leipoa ocellata*) increases water vapor conductance in 85% during incubation due to the embryonic eggshell thinning, about 21% of the total thickness is lost (Booth & Seymour, 1987); additionally these eggs have a 14μm cuticle or accessory layer that occludes the pores; these adaptations respond to the very low oxygen and high carbon dioxide concentrations of the organic rich mound nest and to prevent bacterial penetration (Grellet‐Tinner et al., 2017). Similar to *Apteryx,* the Mallefowl has a very long incubation period (45–50 days) that can become extended (65–70 days) if temperatures are low (Grellet‐Tinner et al., 2017).

Our calculated initial thickness is consistent with previous measurements of the eggshell thickness of unincubated and infertile Kiwi eggs (Calder, 1979; Silyn‐Roberts, 1983). This prediction should be tested, ideally using micro‐CT or another nondestructive X‐ray technique to observe the shell reduction in the same egg as incubation progresses, which for *Apteryx* given its conservation status would be very difficult to do.

Intra‐clutch differences in various eggshell characteristics such as gas conductance (Clark et al., 2010), egg weight (Forbes & Ankney, 1988), volume (Henriksen, 1995), and eggshell thickness (Massaro & Davis, 2005) have been observed in other avian species; this means that comparing fertile and infertile eggs even from the same parents could produce equivocal estimations of eggshell thinning.

Measurements of eggshell thickness and water vapor conductance are usually performed on eggshells from infertile or early incubation eggs; however, this was not possible in this case; hence, hatched eggs were used. It is unknown if the rate of eggshell thinning is the same or not between individuals of the same species and this is a subject that should be revised in future studies.

## CONFLICT OF INTEREST

None declared.

## AUTHOR CONTRIBUTIONS

**David Vieco‐Galvez:** Conceptualization (equal); Data curation (equal); Formal analysis (equal); Funding acquisition (equal); Investigation (equal); Methodology (equal); Project administration (lead); Resources (equal); Visualization (equal); Writing‐original draft (equal); Writing‐review & editing (equal). **Isabel Castro:** Conceptualization (equal); Data curation (equal); Formal analysis (equal); Funding acquisition (equal); Investigation (equal); Methodology (equal); Project administration (equal); Resources (equal); Supervision (lead); Visualization (equal); Writing‐original draft (equal); Writing‐review & editing (equal). **Patrick Morel:** Data curation (equal); Formal analysis (equal); Funding acquisition (equal); Investigation (equal); Methodology (equal); Resources (equal); Supervision (equal); Validation (equal); Visualization (equal); Writing‐original draft (equal); Writing‐review & editing (equal). **Wei Hang Chua:** Conceptualization (supporting); Data curation (supporting); Formal analysis (supporting); Investigation (equal); Methodology (equal); Project administration (equal); Supervision (equal); Visualization (equal); Writing‐original draft (equal); Writing‐review & editing (equal). **Michael Loh:** Investigation (supporting); Methodology (supporting); Visualization (equal).

## Data Availability

The data for this publication is available at OSF: Apteryx eggshell structure https://doi.org/10.17605/OSF.IO/X87DB
